# Antidepressant-like drug effects in juvenile and adolescent mice in the tail suspension test: Relationship with hippocampal serotonin and norepinephrine transporter expression and function

**DOI:** 10.3389/fphar.2013.00131

**Published:** 2013-10-28

**Authors:** Nathan C. Mitchell, Georgianna G. Gould, Corey M. Smolik, Wouter Koek, Lynette C. Daws

**Affiliations:** ^1^Department of Physiology, University of Texas Health Science CenterSan Antonio, TX, USA; ^2^Department of Psychiatry, University of Texas Health Science CenterSan Antonio, TX, USA; ^3^Department of Pharmacology, University of Texas Health Science CenterSan Antonio, TX, USA

**Keywords:** antidepressant, selective serotonin reuptake inhibitor, tricyclic, serotonin transporter, norepinephrine transporter, juvenile, adolescent, depression

## Abstract

Depression is a major health problem for which most patients are not effectively treated. This problem is further compounded in children and adolescents where only two antidepressants [both selective serotonin reuptake inhibitors (SSRIs)] are currently approved for clinical use. Mouse models provide tools to identify mechanisms that might account for poor treatment response to antidepressants. However, there are few studies in adolescent mice and none in juvenile mice. The tail suspension test (TST) is commonly used to assay for antidepressant-like effects of drugs in adult mice. Here we show that the TST can also be used to assay antidepressant-like effects of drugs in C57Bl/6 mice aged 21 (juvenile) and 28 (adolescent) days post-partum (P). We found that the magnitude of antidepressant-like response to the SSRI escitalopram was less in P21 mice than in P28 or adult mice. The smaller antidepressant response of juveniles was not related to either maximal binding (*B*_max_) or affinity (*K*_d_) for [^3^H]citalopram binding to the serotonin transporter (SERT) in hippocampus, which did not vary significantly among ages. Magnitude of antidepressant-like response to the tricyclic desipramine was similar among ages, as were *B*_max_ and *K*_d_ values for [^3^H]nisoxetine binding to the norepinephrine transporter in hippocampus. Together, these findings suggest that juvenile mice are less responsive to the antidepressant-like effects of escitalopram than adults, but that this effect is not due to delayed maturation of SERT in hippocampus. Showing that the TST is a relevant behavioral assay of antidepressant-like activity in juvenile and adolescent mice sets the stage for future studies of the mechanisms underlying the antidepressant response in these young populations.

## INTRODUCTION

Depression is a major public health problem for which most patients are not effectively treated. This problem is further compounded in children and adolescents by limited pharmacological treatment options (Bylund and Reed, 2007). The selective serotonin reuptake inhibitor (SSRI) fluoxetine is currently the only FDA approved treatment for depression in children and adolescents up to 18 years old, and escitalopram is approved for children and adolescents age 12 and older. Exacerbating the situation further, children and adolescents respond poorly to these antidepressants compared with adults (Tsapakis et al., 2008; Hetrick et al., 2009, 2010). Given the high prevalence of adolescent depression, affecting 4–8% of the population with an incidence of 25% by the end of adolescence (Kessler et al., 2001; Bujoreanu et al., 2011) and, of major concern, the high prevalence of suicide in this young population (the third leading cause of death in the 15- to 19-year age group; Reed et al., 2008), there is a clear need to understand the neural mechanisms accounting for these differences between children and adolescents on the one hand and adults on the other, with the hope to uncover targets for the development of more effective treatments. However, despite many reports showing marked differences in the antidepressant response of children and adolescents compared with adults (Bylund and Reed, 2007; Tsapakis et al., 2008; Hetrick et al., 2009, 2010; Hazell and Mirzaie, 2013) there is a paucity of studies investigating the underlying mechanisms. Thus, reasons for the age-dependency of antidepressant response remain poorly understood.

Animal models are needed to examine the mechanisms underlying age-dependent effects of antidepressants. To date, there are only a few preclinical studies of antidepressants in juvenile and early adolescent animals, and most have been conducted using rats. SSRIs were found to reduce time spent immobile in the forced swim test (FST), an index of antidepressant-like activity, in rats as young as postnatal day (P) 21 as well as in adults, whereas blockers of the norepinephrine (NE) transporter, such as the tricyclic antidepressant desipramine (DMI), were ineffective in the FST in rats younger than P28 (Pechnick et al., 2008; Reed et al., 2008). The mechanistic basis for these findings remains to be determined, but is thought to involve the delayed maturation of the NE neurotransmitter system relative to the serotonin (5-HT) system. In terms of the actual drug targets themselves, i.e., the serotonin and norepinephrine transporters (SERT and NET, respectively), information about their expression in juvenile and adolescent animals is sparse. Using quantitative autoradiography, Galineau et al. (2004) reported a triphasic profile for SERT in amygdala and hypothalamus of rats where expression peaked around P21, decreased at P28 and plateaued through P70, the oldest age tested (see also Daws and Gould, 2011). For NET, Sanders et al. (2005) also using autoradiography, reported that expression of NET in some brain regions (e.g., locus coeruleus) was much greater in rats aged P20 than in adults, while in other regions NET expression in P20 rats was either less than (e.g., CA3 region of hippocampus) or similar (e.g., cortex, CA1 and CA2 regions of hippocampus, dentate gyrus) to that of adult rats. Thus, it is not clear from studies in rats, if expression or activity of SERT and NET correlates positively with the emergence of an antidepressant-like response to SSRIs and NET blockers.

Lacking are studies in mice to probe the mechanistic basis underlying differences in antidepressant-efficacy among juveniles, adolescents, and adults. The relative ease with which mice can be genetically manipulated makes them a powerful tool for preclinical research. However, there are few studies that have used adolescent (≥P28) mice to investigate antidepressant-like response (Bourin et al., 1998; David et al., 2001a; Mason et al., 2009) and none that have used mice younger than P28. Although mice have been used to examine the consequences of antidepressant treatment during prenatal, early postnatal and adult periods, juvenile and adolescent periods remain largely unexplored. Likewise, little is known about SERT and NET expression during these juvenile and adolescent periods in mice.

The tail suspension test (TST) is a preclinical test with good predictive validity that has become one of the most widely used models for assessing antidepressant-like activity in adult mice (Cryan et al., 2005). Currently there is only one report of its use in adolescent (P35) mice. Thus, it is unknown if the TST can be used to detect antidepressant-like effects of drugs in early adolescent (P28) and juvenile (P21) mice. The goals of the present study were twofold: first, to examine if the TST can be used to measure antidepressant-like activity in P21 and P28 mice; and second, to begin to examine the relationship between antidepressant-like activity and the expression and affinity of hippocampal SERT and NET in juvenile, adolescent, and adult mice.

## MATERIALS AND METHODS

### ANIMALS

Juvenile (P21), early adolescent (P28), and adult (P62–90) male and female C57Bl/6 mice were obtained from an in house breeding colony (breeding pairs originally obtained from Jackson Lab). Body weights for male mice ranged from 6.6 to 9.8 g for P21, from 9.7 to 18.5 g for P28, and from 23.7 to 42.8 g for adults, and body weights for female mice ranged from 6.3 to 10.0 g for P21, from 10.8 to 15.1 g for P28, and from 19.1 to 31.3 g for adults. Animals were housed in a temperature-controlled (24°C) vivarium maintained on a 14/10-h light/dark cycle (lights on at 07:00, experiments conducted during the light period) in plastic cages (29 cm × 18 cm × 13 cm) containing rodent bedding (Sani-chips, Harlan Teklad, Madison, WI, USA) with free access to food (Rodent sterilizable diet, Harlan Teklad, Madison, WI, USA) and water. After weaning on postnatal day 21, mice were housed in groups of five with same-sex peers. All procedures were conducted in accordance with the National Institute of Health Guide for the Care and Use of Laboratory Animals (Institute of Laboratory Animal Resources, Commission on Life Sciences, National Research Council 1996), and with the Institutional Animal Care and Use Committee, The University of Texas Health Science Center at San Antonio.

### TAIL SUSPENSION TEST

The TST was conducted based on the original description by Steru et al. (1985) [for a review, see (Castagne et al., 2011)]. On the day before testing, mice were moved from the colony room and housed overnight in a holding room adjoining the procedure room. On the test day, mice were placed in the procedure room and allowed 1–2 h to acclimate before receiving an injection of saline vehicle (subcutaneously [sc] or intraperitoneally [ip]), escitalopram (10 mg/kg, sc), or DMI (32 mg/kg, ip). Routes of drug administration were based on results in adult mice reported by Cryan et al. (2005) and Sanchez et al. (2003). Each mouse was tested only once (i.e., not given multiple drugs nor exposed to the TST on multiple occasions). Drugs or saline were injected 30 min before testing. Immediately before testing, the distal end of the tail was fastened to a flat aluminum (2 × 0.3 × 10 cm) bar using adhesive tape at a 90° angle to the longitudinal axis of the mouse tail and the aluminum bar, with a distance of 3–4 cm between the base of the tail and the edge of the bar. A hole opposite the taped end of the bar was used to secure the bar to a hook in the ceiling of a visually isolated white test box (40 cm × 40 cm × 40 cm). Each mouse was suspended by its tail for 6 min, allowing the ventral surface and front and hind limbs to be video-recorded using a digital camera facing the test box. Total time immobile was measured (in seconds) during the entire 6 min test period. Immobility was defined as the absence of initiated movements, and included passive swaying of the body. A mouse was excluded from the experiments if it climbed and held on to its tail or the aluminum bar for a period of 3 s or longer. In the present study no mice aged P21 or P28 were excluded. Approximately 5% of adult mice were excluded. Immobility was scored manually by observers watching the video and who were blind to the treatment. Typically two observers scored each videotape with excellent inter-observer agreement (*r*^2^ > 0.9).

Initial experiments were designed to examine the utility of the TST in juvenile and adolescent mice. Male and female mice were given either a sc or ip injection of saline, corresponding to the route of administration for escitalopram and DMI, respectively. The purpose of these initial experiments was to identify possible effects of age, gender, and route of administration, as well as any interactions among these factors, on immobility in the TST. Age affected basal immobility, and did so in a similar manner in both genders after both routes of administration (see Results). Subsequent experiments investigated the two reference antidepressant drugs, escitalopram (10 mg/kg, sc) and DMI (32 mg/kg, ip), in male mice. Drug doses were selected based on preliminary data obtained in our laboratory that showed these doses to be the lowest to produce maximal effects on immobility in adult C57Bl/6J mice.

### [^3^H]CITALOPRAM AND [^3^H]NISOXETINE SATURATION BINDING IN HIPPOCAMPAL HOMOGENATES

All binding experiments were carried out using tissue from male C57Bl/6 mice.

#### [^3^H]citalopram binding to SERT

Saturation binding of [^3^H]citalopram in membrane homogenate preparations from mouse hippocampi was carried out following the methods of D’Amato et al. (1987) with minor modifications. Briefly, male mice were decapitated, the brain removed and hippocampi collected. Hippocampi from individual mice were homogenized in 25 ml of 4°C 50 mM Tris, 120 mM NaCl, 5 mM KCl buffer (pH 7.4 at 25°C), at 2600 rpm on a Polytron tissue homogenizer (Brinkman Instruments, Westbury, NY, USA). The homogenate was centrifuged for 10 min at 30,600 × *g* at 4°C. The supernatant was discarded, and the pellet re-suspended on ice using a Potter Elvehjem glass and Teflon homogenizer in 25 ml ice-cold buffer. The homogenate was re-centrifuged for 10 min at 30,600 × *g*. The final pellet was re-suspended to yield a protein concentration of approximately 0.5–1.2 μg/μl. Protein was quantified spectrophotometrically on a plate reader (SpectraMax 190, Molecular Devices, Sunnyvale, CA, USA) using Bradford reagent (Sigma, St. Louis, MO, USA). Binding assays were run in triplicate for each hippocampal membrane homogenate preparation. Homogenates were incubated at 25°C for 1 h in buffer (50 mM Tris, 120 mM NaCl, 5 mM KCl) containing 0.1–10 nM [^3^H]citalopram (Perkin Elmer). Non-specific binding was defined by addition of 10 μM sertraline (Pfizer). Incubation was terminated by addition of 4 ml of ice cold buffer and rapid filtration under vacuum onto Whatman GF/B filter paper strips (Brandel, Gaithersburg, MD, USA) pre-soaked in 5% polyethyleneimine (Sigma). Filters were washed twice and radioactivity trapped on the filters was measured by liquid scintillation counting using a Beckman 6500 (Beckman, Brea, CA, USA) with efficiencies of 40–65%. Binding data were analyzed by non-linear regression using GraphPad Prism 5.04.

#### [^3^H]nisoxetine binding to NET

Saturation binding of [^3^H]nisoxetine in membrane homogenate preparations from mouse hippocampi was carried out following the methods of Tejani-Butt (1992). Hippocampal homogenate preparation for [^3^H]nisoxetine binding was as described for [^3^H] citalopram binding, except that hippocampi were pooled from two mice to yield protein concentrations of 1.0–1.7 μg/μl, the buffer was pH 7.4 at 4°C and the final washed pellet was re-suspended in 50 mM Tris, 300 mM NaCl, 5 mM KCl (pH 7.4 at 4°C). Binding assays were carried out for 4 h at 4°C in 50 mM Tris, 300 mM NaCl, 5 mM KCl at the same volumes used for [^3^H] citalopram binding. [^3^H] nisoxetine concentration ranged from 0.5 to 30 nM for the saturation assays. Non-specific binding was defined with 10 μM mazindol (Sigma, St Louis, MO, USA). Data collection and analysis were the same as described for [^3^H]citalopram binding.

### STATISTICAL ANALYSIS

Statistical analyses were performed using Prism 5.04 (GraphPad, San Diego, CA, USA) and NCSS 2007 (Kaysville, UT, USA). TST data were analyzed using ANOVA, followed by Tukey’s multiple comparison tests. Binding data were analyzed using Kruskal–Wallis test because of significant differences in standard deviations among age groups (Bartlett’s test). All data are expressed as mean ± standard error of the mean (SEM), and *P* < 0.05 was considered statistically significant.

### DRUGS

Escitalopram oxalate [Shanco International Inc. (Hazlet, NJ, USA)] and DMI hydrochloride [Sigma-Aldrich (St. Louis, MO, USA)] were dissolved in physiological saline. Escitalopram was injected sc at doses expressed as base per kilogram body weight (Sanchez et al., 2003). DMI was injected ip at doses expressed as salt per kilogram body weight. The injection volume was 10 ml/kg.

## RESULTS

### USE OF THE TST IN JUVENILE AND ADOLESCENT MICE

**FIGURE 1:**
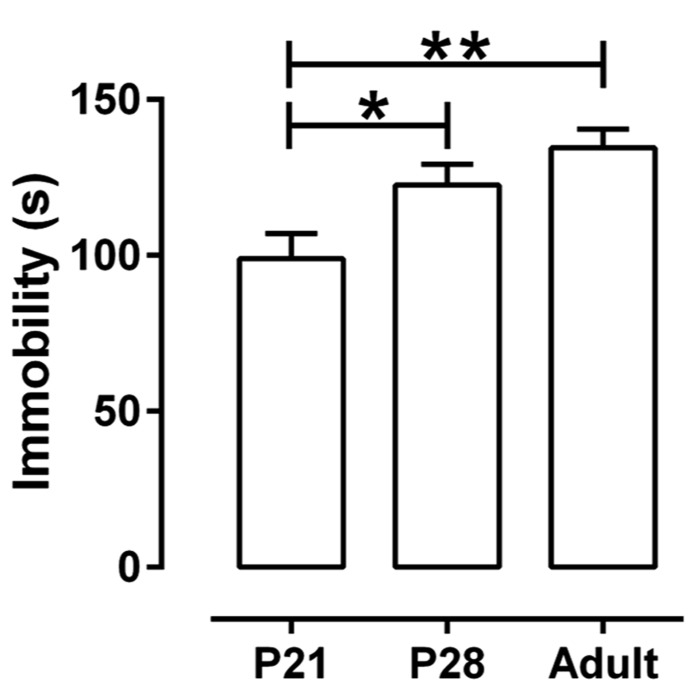
**Immobility time in theTST as a function of age.** Juvenile mice (P21) spent significantly less time immobile than either adolescent (P28) or adult mice (*P* > 62). Each age group consisted of 40 animals treated with saline (20 males and 20 females, half of them treated sc and the other half ip). Because there were neither main nor interaction effects of gender and route of administration, data are shown collapsed with age as the only factor. *P < 0.05, **P < 0.01 (Tukey *post hoc* test). Data are mean and SEM.

The TST is a preclinical test with good predictive validity that is widely used to detect antidepressant-like activity (Cryan and Holmes, 2005; Cryan et al., 2005), and that has been used in mice as young as P35 (Mason et al., 2009). Antidepressant-like activity in this test is defined by the ability of a drug to reduce the time a mouse spends immobile. We first examined if the TST could be used in P21 and P28 mice, given the possibility that young mice may display so little baseline immobility that the effect of a drug to reduce immobility further may not be detectible. To this end, six separate groups of mice (male and female, *n* = 20 of each gender and each age, P21, P28, or adult) received an injection of saline either sc or ip (*n* = 10 of each gender and age receiving saline sc, and *n* = 10 of each gender and age receiving saline ip) and time spent immobile in a 6 min test was quantified. There was no significant effect of gender or route of administration on immobility time, a finding that is consistent with reports for adult mice (e.g., David et al., 2001b; Jones and Lucki, 2005; Andreasen and Redrobe, 2009). There was however, a significant effect of age [*F*(2, 119) = 6.63, *P* < 0.0025]. Because there were no significant interactions among age, gender, and route of administration, data were collapsed with age as the only variable (**Figure 1**). P21 mice spent significantly less time immobile (99 ± 8 s) than P28 (123 ± 7 s) or adult mice (135 ± 6 s), and P28 mice did not differ significantly from adults. A factor that might contribute to reduced immobility time in P21 mice is their smaller size. Regression analyses of immobility time as a function of body weight for all ages and both genders, inclusive, revealed an overall positive correlation (*r* = 0.24, *P* < 0.01). However, regression analyses within each age group revealed no significant correlation between body weight and immobility time (P21, *r* = -0.12, *P* = 0.46; P28, *r* = 0.23, *P* = 0.11; adult, *r* = 0.05, *P* = 0.66, data not shown).

These data show that juvenile and adolescent mice spend sufficient time immobile in the TST that detection of a drug effect to decrease immobility should be possible. To test this, juvenile and adolescent mice were treated acutely with either escitalopram (sc) or DMI (ip), two antidepressants known to produce robust effects in the TST in adult mice (Sanchez et al., 2003; O’Leary et al., 2007).

### REFERENCE ANTIDEPRESSANTS REDUCE IMMOBILITY ACROSS AGES

**FIGURE 2:**
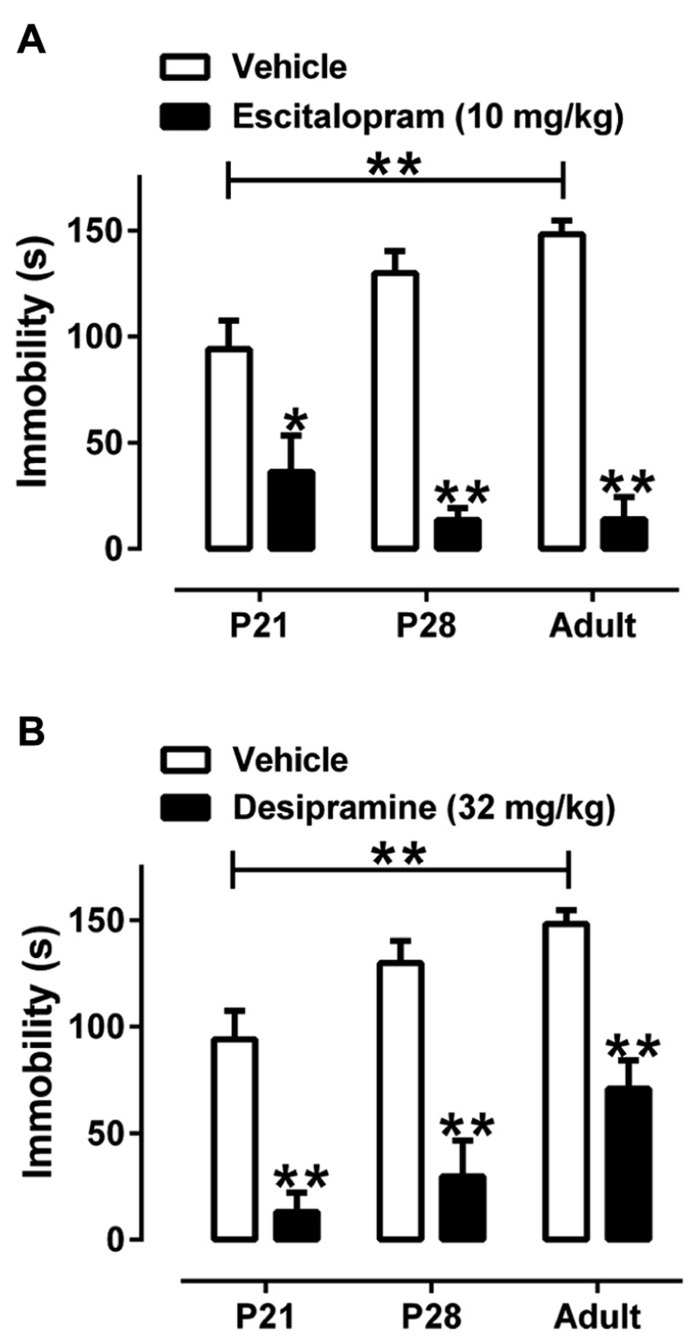
**Effect of escitalopram and DMI to reduce time spent immobile in the TST in male juvenile, adolescent, and adult mice.** **(A)** Escitalopram (10 mg/kg, sc) significantly reduced immobility time in all age groups. There was a significant interaction between age and treatment revealing that escitalopram is less effective in reducing immobility time in P21 mice, compared with P28 or adult mice. **(B)** DMI (32 mg/kg, ip) significantly reduced immobility time in all age groups. Sample sizes were as follows: P21, *n* = 38 (20 saline, 8 escitalopram, 10 DMI); P28, *n* = 37 (20 saline, 8 escitalopram, 8 DMI); adult mice *n* = 39 (20 saline, 10 escitalopram, 9 DMI). There was no main effect of route of saline administration, so saline data were collapsed with age and drug as the only factors; two-way ANOVA with Tukey’s *post hoc* tests, *P < 0.05, **P < 0.01. Data are mean and SEM.

Escitalopram (10 mg/kg, sc) and DMI (32 mg/kg, ip) reduced immobility time in the TST in all age groups [*F*(1, 51) = 66.45, *P* < 0.01] (**Figure 2**). However, the extent to which they decreased immobility differed among the age groups. For escitalopram there was a significant interaction between treatment and age [*F*(2, 51) = 5.08, *P* < 0.01] because escitalopram reduced immobility less in P21 mice than in P28 and adult mice. For DMI there was a significant effect of age [*F*(2, 51) = 7.14, *P* < 0.01] and DMI tended to reduce immobility more in P21 and P28 mice than in adults, but the interaction between treatment and age did not reach statistical significance. Sample sizes for saline-, escitalopram-, and DMI-treated mice, respectively, were 20, 8, and 10 for mice aged P21; 20, 8, and 8 for mice aged P28, and 20, 10, and 9 for adult mice. The larger sample size for saline-treated mice is due to pooling data from male mice injected with saline sc (*n* = 10) and ip (*n* = 10) for each age. These results show that the TST can be used to examine antidepressant-like drug effects in mice as young as P21. Next we investigated the relationship between the antidepressant-like effects of escitalopram and DMI and the expression of their targets, the SERT and NET, respectively, in hippocampus of P21, P28, and adult mice.

### [^3^H]CITALOPRAM AND [^3^H]NISOXETINE SATURATION BINDING IN HIPPOCAMPUS AS A FUNCTION OF AGE

**FIGURE 3:**
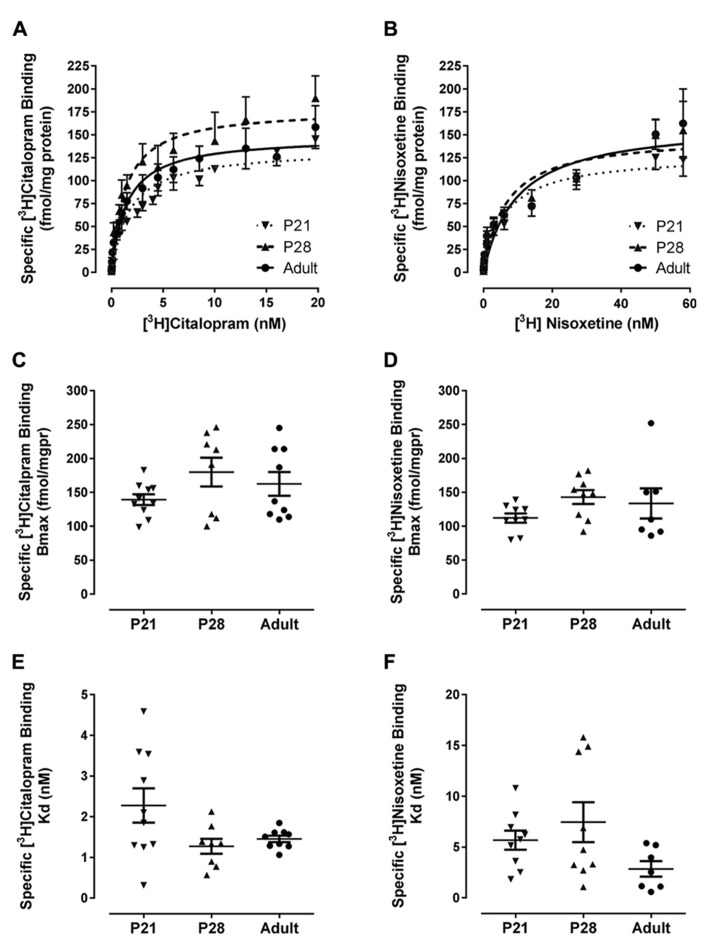
**Specific binding of [^3^H]citalopram to SERT and [^3^H]nisoxetine to NET in hippocampal membrane homogenates from male P21(▼) P28 (▲) and adult (•) mice.** Membrane preparations were incubated with increasing concentrations of [^3^H]citalopram or [^3^H]nisoxetine. Non-specific binding was defined in the presence of 10 μM sertraline or 10 μM mazindol, respectively. Specific binding was obtained by subtracting non-specific binding from total binding at each ligand concentration. **(A,B)** Show saturation binding isotherms for binding of [^3^H]citalopram or [^3^H]nisoxetine, respectively. *B*_max_ and *K*_d_ values for [^3^H]citalopram to SERT are summarized in **(C,E)**, and for [^3^H]nisoxetine binding to NET in **(D,F)**, respectively. For [^3^H]citalopram sample sizes were as follows: P21 *n* = 10, P28 *n* = 8, adult *n* = 9; and for [^3^H]nisoxetine, P21 *n* = 9, P28 *n* = 9, adult *n* = 7. Data are mean and SEM.

As shown in **Figures 3A,C,E** and **Table 1**, [^3^H]citalopram saturation binding in mouse hippocampal homogenates revealed no significant difference in maximal binding (*B*_max_) or affinity (*K*_d_) values among P21 (*n* = 10), P28 (*n* = 8), and adult (*n* = 9) male mice. Likewise, [^3^H]nisoxetine saturation binding in mouse hippocampal homogenates revealed no significant difference in *B*_max_ or *K*_d_ values among P21 (*n* = 9), P28 (*n* = 9), and adult (*n* = 7) male mice (**Figures 3B,D,F**; **Table 1**). Of note is the greater variability in *K*_d_ values for [^3^H]citalopram binding in P21 mice, compared with their P28 and adult counterparts (*P* < 0.001, Bartlett’s test). Similarly, the variance of the *K*_d_ values for [^3^H]nisoxetine binding in young mice was greater than in adults (*P* < 0.01, Bartlett’s test), suggesting that these young ages may represent a transitional period where SERT and NET are shifting toward the functional activity state of adult SERT and NET.

**Table 1:** Summary of *B*_**max**_ and *K*_d_ values for [^**3**^H]citalopram binding to SERT and [^**3**^H]nisoxetine binding to NET in male P21, P28, and adult mice.

	P21	P28	Adult
**[^3^H]Citalopram**
*B*_max_ (fmol/mgpr)	139 ± 8	180 ± 21	163 ± 17
*K*_d_ (nM)****	2.3 ± 0.4	1.3 ± 0.2	1.5 ± 0.1
**[^3^H]Nisoxetine**
*B*_max_ (fmol/mgpr)	112 ± 7	143 ± 10	136 ± 23
*K*_d_ (nM)	5.7 ± 0.9	7.4 ± 2.0	2.5 ± 0.8

*[^*3*^H]citalopram binding *n* = 8–10 per group where hippocampi were from one mouse per assay. For [^3^H]nisoxetine binding *n* = 7–9 per group, but where each assay was from pooled hippocampi from two mice. For each ligand, there were no significant differences in *B*_max_ or *K*_d_ values among ages (Kruskal–Wallis). Data are mean and SEM.*

### RELATIONSHIP BETWEEN ANTIDEPRESSANT-LIKE EFFECT AND SATURATION BINDING WITH [^3^H]CITALOPRAM AND [^3^H]NISOXETINE IN HIPPOCAMPUS ACROSS AGE GROUPS

**FIGURE 4:**
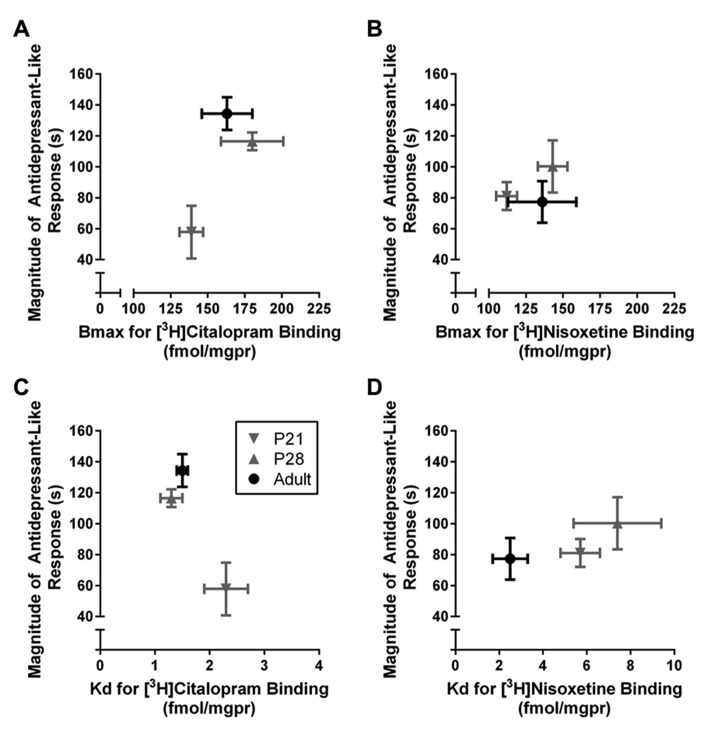
**Relationship between antidepressant-like response and hippocampal SERT and NET expression and affinity in P21, P28, and adult male mice.** The magnitude of antidepressant-like response to the SERT blocker, escitalopram, increased with age but neither *B*_max_ **(A)** nor *K*_d_ **(C)** values for [^3^H]citalopram binding to SERT varied significantly with age. The magnitude of antidepressant-like response to the NET blocker, DMI, as well as *B*_max_ **(B)** and *K*_d_ **(D)** values for [^3^H]nisoxetine binding to NET remained relatively constant across ages. Data are re-plotted from **Figure 2** and **Table 1**. TST data from **Figure 2** are plotted on the *y*-axis as “magnitude of antidepressant-like response,” defined as the immobility time for individual drug-treated mice subtracted from the mean immobility time of same aged saline-treated mice; the larger the number, the greater the antidepressant-like effect. Sample sizes are the same as reported in legends to **Figures 2,3** and **Table 1**. Data are mean and SEM.

Data from **Figure 2** and **Table 1** are plotted in Figure 4 and illustrate the relationship, or lack thereof, between the ability of escitalopram and DMI to produce antidepressant-like effects in the TST and the expression and affinity values for hippocampal SERT (**Figures 4A,C**) and NET (**Figures 4B,D**). TST data plotted in **Figure 4** are the immobility times for each individual escitalopram- or DMI-treated mouse, subtracted from the mean value for immobility time of the same age saline-treated mice (i.e., data from **Figure 2**). This difference provides a measure of the magnitude of antidepressant-like response that takes into account the difference in immobility times among saline-treated mice of different ages. The ability of escitalopram to produce antidepressant-like effects in the TST increased with age, but was not associated with parallel increases in either *B*_max_ (**Figure 4A**) or affinity of SERT for [^3^H]citalopram (i.e., smaller *K*_d_ values; **Figure 4D**). The ability of DMI to produce antidepressant-like effects did not change significantly across age groups. Likewise, *B*_max_ and *K*_d_ values for [^3^H]nisoxetine binding to NET did not vary significantly as a function of age.

## DISCUSSION

The major findings of the present study are first, that the SSRI escitalopram and the NET blocker DMI produced antidepressant-like effects in mice as young as P21, the youngest age tested; second, that the magnitude of antidepressant-like response to escitalopram increased with age but was not paralleled by increasing expression or affinity of hippocampal SERT; and third that the magnitude of antidepressant-like response to the tricyclic, DMI, as well as expression and affinity of hippocampal NET, did not differ significantly among P21, P28, and adult mice. These findings support the utility of juvenile mice to study antidepressant-like activity of drugs. Moreover, our finding that juvenile mice are less sensitive to the antidepressant-like effect of escitalopram than adults, parallels clinical data reporting that, compared to adults, children have a relatively poor therapeutic response to the SSRIs, fluoxetine, and escitalopram, the only two FDA approved antidepressants for this young population.

In rodents, postnatal days 21–27 are considered the juvenile period and postnatal days 28–42 equivalent to early adolescence (Spear, 2000; Bylund and Reed, 2007). To date only a few studies have investigated antidepressant-like activity of drugs in adolescent mice and none have studied juvenile mice. Adolescent mice (P28 or P35) were found to be sensitive to the antidepressant-like effect of both SSRIs and tricyclics in the FST (Bourin et al., 1998; David et al., 2001a,b; Mason et al., 2009). Only one study has used the TST to investigate antidepressant-like activity in adolescent mice (P35) and, as for studies using the FST, found that both SSRIs and tricyclics were effective in reducing immobility time. Consistent with these studies, we also found that both SSRI and tricyclic classes of antidepressant effectively reduced immobility time of adolescent (P28) and adult mice in the TST. Likewise, our findings in P28 and adult mice are in good agreement with reports in P28 and adult rats, where both SSRIs and tricyclic antidepressants were effective in producing antidepressant-like activity in the FST (Reed et al., 2008).

To our knowledge, this is the first report of antidepressant-like activity in P21 mice. We found that the SSRI, escitalopram, produced antidepressant-like activity in these mice; however the magnitude of this effect was less than that in adolescent (P28) or adult mice. Similarly, studies using rats aged P21 in the FST, found them to be sensitive to the antidepressant-like effects of SSRIs, including escitalopram (Reed et al., 2008). However, in this study a head-to-head comparison with P28 and adult rats was not included, making it difficult to draw conclusions as to whether the magnitude of antidepressant-like effect was less in P21 rats, compared to P28 or adult rats. Based on the present studies, where P21, P28, and adult mice were compared head-to-head, it is clear that the magnitude of escitalopram to produce antidepressant-like effects in P21 mice is less than in adult mice.

We also found that P21 mice were sensitive to the antidepressant-like effect of the tricyclic, DMI. Unlike our finding for escitalopram, the magnitude of antidepressant-like effect of DMI was similar among P21, P28, and adult mice. This finding contrasts with studies using rats, where Reed et al. (2008) found P21 rats to be insensitive to tricyclics, including DMI. Potential reasons for these differences include species (rat versus mouse), behavioral test (FST versus TST), drug dose, and route of administration. For example, the highest dose of DMI tested in studies using P21 rats was 20 mg/kg (ip; Reed et al., 2008), whereas in our studies using mice, the dose was 32 mg/kg (ip). Thus, our ability to detect antidepressant-like effects of DMI in mice as young as P21 may result in part from using a higher dose. Certainly, pharmacokinetic differences between species and across ages are also a consideration.

In the clinical setting a key difference between adult and pediatric depression is response to pharmacotherapy (Hazell et al., 1995; Kratochvil et al., 2006; Bridge et al., 2007; Bylund and Reed, 2007; Hazell and Mirzaie, 2013). The present studies using mice, as well as published reports using rats, show that like humans, juvenile mice, and rats respond differently to antidepressant drugs. While there are some apparent discrepancies in reported findings, particularly those relating to the emergence of antidepressant-like activity of DMI, there are numerous factors that may account for these; some of which have been touched on already (e.g., dose, species, test). With regard to the clinical setting, it is important to keep in mind that therapeutic benefit is also contingent upon tolerability of the drug. Thus, although tricyclics are not approved for use in children and adolescents, data from clinical trials have been mixed, with some studies reporting that tricyclics lowered depression scores in adolescents, while others found tricyclics to be therapeutically ineffective (Hazell and Mirzaie, 2013). However, consistent with the mechanism of action of tricyclic antidepressants, these drugs were more likely than placebo to produce adverse side effects, including vertigo, tremor, low blood pressure, and dry mouth. Thus, due to inconclusive demonstrations of therapeutic benefit in young humans, and the possibility of harmful side effects, or increased sensitivity to adverse side-effects in this patient population, tricyclic antidepressants are not prescribed for children and adolescents.

The key finding from the present study is that it is possible to detect antidepressant-like activity of drugs in mice as young as P21. This finding opens the door for studies geared to understanding the mechanisms underlying the relatively poor therapeutic response of young humans to SSRIs, which in turn paves the way for identifying treatments with improved therapeutic efficacy. It is worth emphasizing that essentially nothing is known about the mechanisms of antidepressant activity in juvenile and adolescent mice. Rat studies have led the way, but even then, knowledge is not extensive (for review, see Bylund and Reed, 2007) with many unknowns remaining. For example, the effect of antidepressants can be dependent on relative rates of neurotransmitter synthesis and it is not yet known if the activity of neurotransmitter synthesizing enzymes (e.g., tryptophan hydroxylase, tyrosine hydroxylase) varies during these postnatal periods in mice. Here, we began to investigate possible mechanisms underlying the divergent response of juvenile mice to SSRIs and tricyclic antidepressants by first quantifying the expression and affinity of their target proteins, SERT and NET, in hippocampus.

Hippocampus was selected for these initial studies given its importance in mood and antidepressant drug effects (Campbell and McQueen, 2004). In rats the delayed emergence of antidepressant-like activity of tricyclics is thought to be related to delayed maturation of the noradrenergic system compared with the serotonergic system (Murrin et al., 2007). As far as NET is concerned, the primary target of DMI, its expression during postnatal development in rat brain, measured using quantitative autoradiography, is age and brain region dependent. NET expression increases rapidly across brain regions between P10 and P15, and attains adult levels in some regions (e.g., cortex, CA1 and CA2 regions of hippocampus, dentate gyrus, amygdala, striatum) that are maintained into adulthood; in others regions (e.g., CA3 region of hippocampus) NET expression attains adult levels at P15, but then decreases at P20 before returning to adult levels at P25 (Sanders et al., 2005). To date there are no reports on the development of expression of NET in mouse brain over the postnatal ages studied here. Our data show that in hippocampus, NET expression was similar in P21, P28, and adult mice. Likewise, the affinity of NET for [^3^H]nisoxetine was similar across these ages. Data from adult mouse hippocampus presented here are in general agreement with those reported by others [e.g., *B*_max_ 127 ± 5 fmol/mgpr and *K*_d_ 0.7 ± 0.05 nM, C57Bl/6 male mice (Csölle et al., 2013)], and in adult rat cerebral cortex [*B*_max_ 97 ± 12 fmol/mgpr and *K*_d_ 0.8 ± 0.11 nM (Tejani-Butt, 1992)]. Our data are also consistent with the quantitative autoradiography measures of NET in rat hippocampus reported by Sanders et al. (2005), where, with the exception of CA3 region, NET expression did not vary across juvenile, adolescent, and adult ages. Given that we carried out saturation binding assays in homogenates taken from whole hippocampus it would be unlikely we would detect any age-dependent changes in a sub-region of hippocampus (such as CA3). At the expense of anatomical resolution, our saturation binding approach afforded a measure of transporter affinity, which to our knowledge has not been previously reported for P21 or P28 mouse or rat. Our findings in mouse hippocampus show that NET expression and affinity are at adult levels by P21. Given that the antidepressant-like effects of DMI in the TST were also similar among juvenile, adolescent, and adult mice, it appears that NET expression and affinity in hippocampus parallels DMI’s antidepressant efficacy in mice. It must be recognized, however, that this does not rule out the possibility that DMI’s antidepressant-like activity in the TST depends on NET expression in other regions. Further studies are needed to determine the brain region(s) and mechanisms (e.g., transporters, receptors) that mediate antidepressant-like behavioral activity in the TST following administration of NET blockers, and how this may vary with age.

Even less is known about the postnatal development of expression and affinity of SERT in mice. Quantitative autoradiography studies to date indicate that in rats, SERT expression reaches adult levels between birth and P21 (Zhou et al., 2000; Galineau et al., 2004; Bylund and Reed, 2007). Consistent with these findings in rats, we found that SERT expression in hippocampus of P21 and P28 mice was similar to that in adults. We also found that the affinity of hippocampal SERT for [^3^H]citalopram was equivalent among P21, P28, and adult mice. To our knowledge there are only two reports of [^3^H]citalopram binding using adult C57Bl/6 mouse hippocampal homogenate preparations. Our group previously reported values in good agreement with those reported here [*B*_max_ 171 ± 20 fmol/mgpr; *K*_d_ 1.1 ± 0.2 nM (Gould et al., 2011)]. Another group reported a higher *B*_max_ (555 ± 35 fmol/mgpr) but a similar *K*_d_ (1.2 ± 0.1 nM; Csölle et al., 2013). Of note in the present study, even though statistical analyses did not reveal significant differences in affinity of hippocampal SERT for [^3^H]citalopram among ages, the variance of the *K*_d_ values differed significantly among ages. As is clear in **Figure 3E**, *K*_d_ values varied from 0.3 to 4.6 nM in juveniles, 0.6 to 2.1 nM in adolescents and 1.0 to 1.8 nM in adults. Thus, the spread in *K*_d_ values dropped from 4.3 nM in P21 mice, to 1.5 nM in P28 mice and to 0.8 nM in adult mice. These data suggest that juvenile and adolescent periods may be critical periods in development where, although the density of SERT is at adult expression levels, the functional activity (affinity) of SERT is undergoing a transition to that of the adult. In the case of the present data, approximately half of P21 mice had *K*_d_ values in line with those of adults, and the remainder had *K*_d_ values two or more fold greater (i.e., lower affinity for [^3^H]citalopram). Based on these initial data, it is tempting to speculate that this variability in when the “switch” from juvenile to adult SERT affinity occurs, accounts in part for the variability in individual response to SSRIs in pediatric depression.

These studies are, to our knowledge, the first to obtain *B*_max_ and *K*_d_ values for [^3^H]citalopram binding to SERT and [^3^H]nisoxetine binding to NET, two of the most prominent targets of currently available antidepressant drugs, in juvenile and adolescent mice, and the results are in agreement with the few existing reports from adult mice (Gould et al., 2011; Csölle et al., 2013). The present findings raise the possibility that, although SERT expression may be at or near adult levels in P21 mice, the large variability in affinity state of SERT for SSRIs may account, at least in part, for the lower clinical effectiveness of SSRIs in children. Showing that the TST is a relevant behavioral assay of antidepressant-like activity in juvenile (P21) and adolescent (P28) mice, sets the stage for future studies of the mechanisms underlying the antidepressant response in these young populations.

## Conflict of Interest Statement

The authors declare that the research was conducted in the absence of any commercial or financial relationships that could be construed as a potential conflict of interest.
